# Reinterpreting Behavioral Receptive Fields: Lightness Induction Alters Visually Completed Shape

**DOI:** 10.1371/journal.pone.0062505

**Published:** 2013-06-04

**Authors:** Brian P. Keane, Hongjing Lu, Thomas V. Papathomas, Steven M. Silverstein, Philip J. Kellman

**Affiliations:** 1 Center for Cognitive Science, Rutgers University, New Brunswick, Piscataway, New Jersey, United States of America; 2 Department of Psychiatry, the University of Medicine and Dentistry of New Jersey–Robert Wood Johnson Medical School, Piscataway, New Jersey, United States of America; 3 University Behavioral HealthCare, University of Medicine and Dentistry of New Jersey, Piscataway, New Jersey, United States of America; 4 Department of Psychology, University of California Los Angeles, Los Angeles, California, United States of America; 5 Department of Biomedical Engineering, Rutgers University, New Brunswick, Piscataway, New Jersey, United States of America; University of Leuven, Belgium

## Abstract

**Background:**

A classification image (CI) technique has shown that static luminance noise near visually completed contours affects the discrimination of fat and thin Kanizsa shapes. These influential noise regions were proposed to reveal “behavioral receptive fields” of completed *contours*–the same regions to which early cortical cells respond in neurophysiological studies of contour completion. Here, we hypothesized that 1) influential noise regions correspond to the *surfaces* that distinguish fat and thin shapes (hereafter, key regions); and 2) key region noise biases a “fat” response to the extent that its contrast polarity (lighter or darker than background) matches the shape's filled-in surface color.

**Results:**

To test our hypothesis, we had observers discriminate fat and thin noise-embedded rectangles that were defined by either illusory or luminance-defined contours (Experiment 1). Surrounding elements (“inducers”) caused the shapes to appear either lighter or darker than the background–a process sometimes referred to as lightness induction. For both illusory and luminance-defined rectangles, key region noise biased a fat response to the extent that its contrast polarity (light or dark) matched the induced surface color. When lightness induction was minimized, luminance noise had no consistent influence on shape discrimination. This pattern arose when pixels immediately adjacent to the discriminated boundaries were excluded from the analysis (Experiment 2) and also when the noise was restricted to the key regions so that the noise never overlapped with the physically visible edges (Experiment 3). The lightness effects did not occur in the absence of enclosing boundaries (Experiment 4).

**Conclusions:**

Under noisy conditions, lightness induction alters visually completed shape. Moreover, behavioral receptive fields derived in CI studies do not correspond to contours *per se* but to filled-in surface regions contained by those contours. The relevance of lightness to two-dimensional shape completion supplies a new constraint for models of object perception.

## Introduction

**Figure 1:**
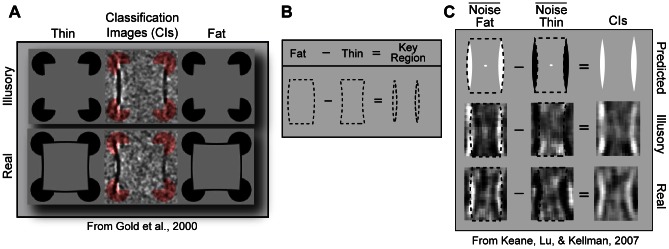
Hypothesis and motivations for the experiments. (A) When subjects discriminated noise-corrupted fat and thin rectangles, noise near real and illusory contours correlated with response. (Modified from a figure courtesy of Jason Gold) (B) The present hypothesis is that correlated noise pixels correspond not to contours but to the *surfaces* that distinguish the fat and thin shapes–the “key regions”. (C) Component images from Keane, Lu, and Kellman (2007) are shown for the illusory and real conditions along with superimposed fat and thin shapes. There is one component image for the fat response and one for the thin response. Each image is the summation of the average noise fields for correct and incorrect trials. The component images suggest that key region pixels biased a fat and thin response by being light and dark, respectively. The final CIs, which result from a simple subtraction, reveal that noise pixel contrast within the key regions positively correlated with a fat response and negatively correlated with a thin response (NB: Images in 1 C are of opposite polarity to Gold et al. (2000), only because in that study the subtraction sequence was reversed).

The neural mechanisms that implement completion include superficial and deep layers of V2 and V1 [1]. Recent work has suggested that the receptive fields for these cells can be revealed behaviorally through a technique termed “classification imaging” [2]–[6]. In an influential study, observers discriminated “fat” and “thin” rectangles, the edges of which were luminance-defined (“real”) or completed (“illusory”; see Figure 1). The rectangles were embedded in a new luminance noise field on each trial and correlations were calculated (across trials) between noise contrast and observer response for each pixel in a noise field. The resulting map of correlations–the classification image–revealed that the noise near illusory contours correlated with response about as much as the noise near real contours. These correlated noise regions were thought to specify the path of completion–the same regions to which cortical cells respond in single-unit studies [7]–[8].

In the current investigation, we hypothesized that influential noise regions in the fat/thin task correspond not to contours *per se*, but to *surfaces* that distinguish the discriminated shapes–hereafter, the *key regions*, (see Figure 1B). On our account, key region noise biases a fat response (and *against* a thin response) to the extent that its achromatic color matches that of the filled-in surface. (Footnote: Correlations between noise pixel contrast and observer response can be stated in different, but logically equivalent, ways. A bias in favor of a fat response is equivalent to a bias against a thin and if lighter noise biases a fat response, then darker noise biases a thin response. Etc.) To motivate the hypothesis, the average noise fields (component images) for a thin and fat response are shown separately for real and illusory shapes [9] (see Figure 1C). The images show that the influential noise–that is, the dark and light areas–largely occupied the key regions. This noise was on average lighter than the background when subjects responded “fat”, and darker than the background when subjects responded “thin”. Since the inducers (ovals) always had negative (Weber) contrast and since a figure surface tends to be lightened in these cases [10], we speculated that lighter key region noise biased a fat response because it resembled the filled-in surface color. To state it more explicitly, as the noise became lighter and therefore closer in appearance to the surface, the key regions were assimilated into the rest of the figure, increasing the likelihood of a fat response. As the key region noise became darker and therefore dissimilar to the rest of the shape, the key regions were viewed as separate from the figure, and a thin response became more likely. Two predictions follow. One is that the relationship between noise influence and response should *reverse* if lightness induction causes shapes to appear darker (rather than lighter) than the background, as would happen with positive contrast inducers. In this case, darker key region noise would be assimilated into the darkened shape, rendering a “fat” response more probable. Another prediction is that if the inducers were half dark and half light (mixed)–so that the figure surface were neither darker nor lighter than the background–then the color of the key region noise would become irrelevant, in which case there would be no consistent relationship between noise and response. Our predictions, if confirmed, would give a new interpretation of CIs and show that the way that the visual system delimits a shape depends on how it fills-in achromatic surface color.

## Approach

**Figure 2:**
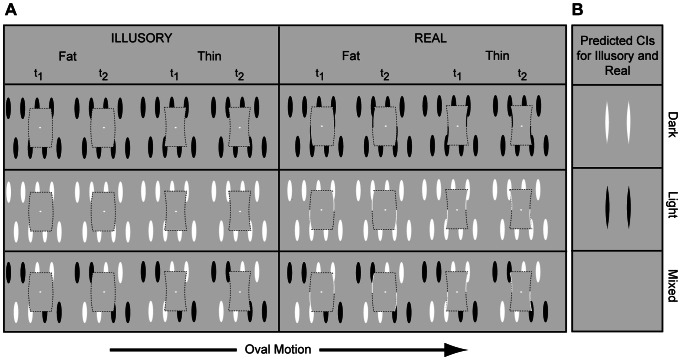
Stimuli and predictions for Experiment 1. (A) On each trial, a fat or thin gray stationary rectangle became visible by occluding horizontally translating ovals. (Dotted shapes are shown for illustration only). Ovals are shown translating to the right, but could translate in either direction. Ovals were either all dark, all light, or half dark and half light (mixed). The top and bottom parts of the rectangles were connected by luminance-defined (real) or completed (illusory) contours, and different parts of these contours appeared at different points in time (t_1_ and t_2_). In the mixed polarity condition, the added real contours were light; in other cases, they were the same contrast as the surrounding ovals. Static luminance noise is not shown above, but encompassed the entire shape and was freshly presented for each trial. (See Movies S1–S12 for dynamic versions of stimuli). (B) Key region pixel contrast was expected to positively and negatively correlate with a fat response when inducers were dark and light, respectively. No correlations were expected for the mixed-polarity condition. No specific predictions were made for non-key regions, which are shown above as mean gray.

Our set-up was similar to that of Gold, Murray, Bennett, & Sekuler (2000), except that a fat or thin rectangle became visible as a result of eight ovals passing behind it (Figure 2A; see also [9]). A spatiotemporal version of the fat/thin task was chosen because interpolation may be stronger and cause a greater reliance on filling-in regions when edge information must be combined over time [11]–[12]. Spatiotemporal displays are also useful for creating minimally lightened surfaces: In our mixed polarity condition, dark and light ovals contributed equally to the appearance of each inducing edge, so that there was strong contour completion without substantive changes in surface lightness (see Methods).

**Figure 3:**
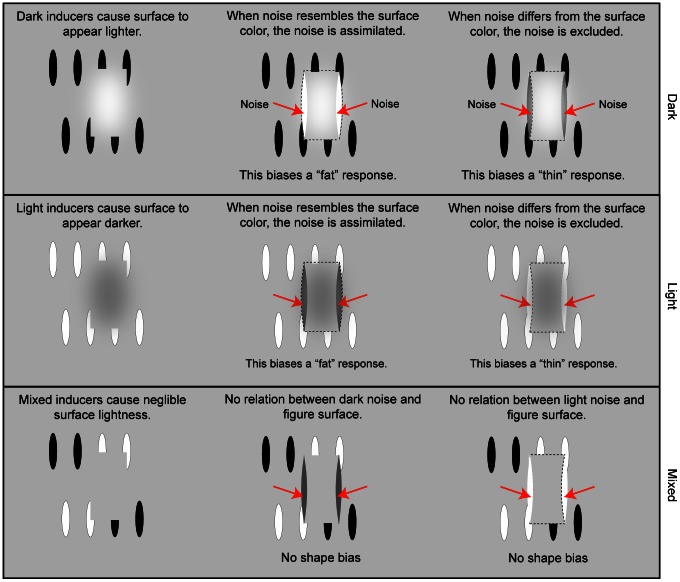
A graphical explanation of the predictions for the inducer types (dark, light, and mixed). The red arrows indicate the location of the highly influential key regions.

Our hypothesis was tested over the course of four experiments. In Experiment 1, ovals were either: all dark, to create a lightened figure surface; all light, to produce a darkened figure surface; or half dark and half light (mixed), to minimize lightness induction. Discriminated contours were either real or illusory (see Figure 2A). The real contours were the same contrast as the ovals, except for the mixed condition, where they were the same contrast as the light ovals. A Bayesian adaptive algorithm (QUEST) adjusted signal (oval) contrast to maintain 70% accuracy [13]. CIs were computed for each of the six conditions [4]. Two “multi-session” observers each performed 10000 trials per condition over the course of several weeks; 50 additional “one-session” observers each performed 200 trials/condition over the course of one hour. The predictions, which are illustrated in Figure 2B and explained more fully in Figure 3, are as follows: i) when inducers are dark, key region noise pixel contrast will *positively* correlate with a fat response producing light CI features; ii) when inducers are light, key region noise will *negatively* correlate with a fat response producing dark CI features; and iii) when the inducing edges are light and dark over the course of a trial, there will be little, if any, correlation between noise and response (producing uniform gray CIs).

## Results

**Figure 4:**
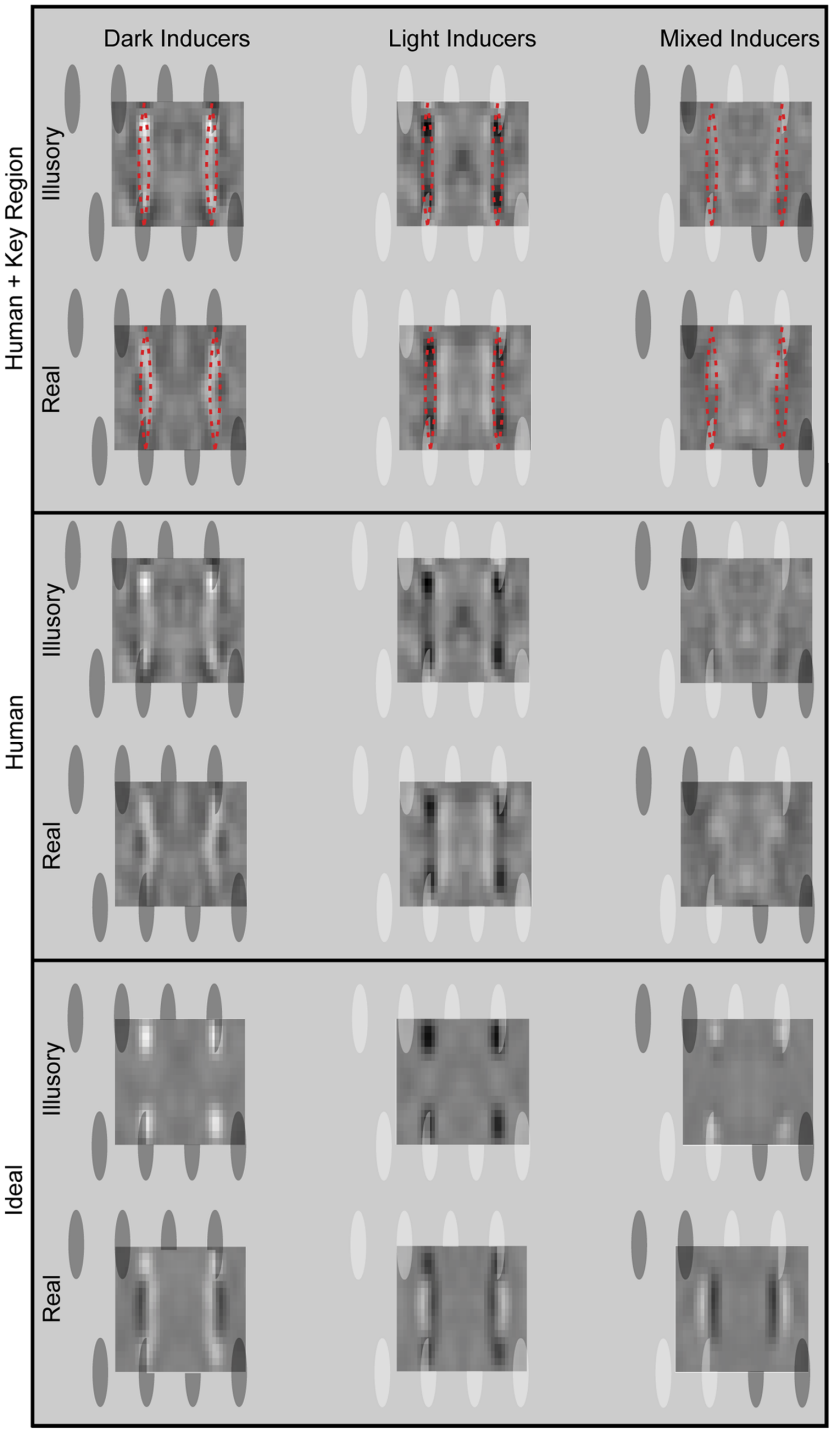
Convolved CIs for human and ideal observers computed from 30,000 and 3,000 trials per condition, respectively. The superimposed translucent ovals show the position of an average rectangle (neither fat nor thin). Human CIs are shown with and without superimposed key regions (dotted red lines).

**Figure 5:**
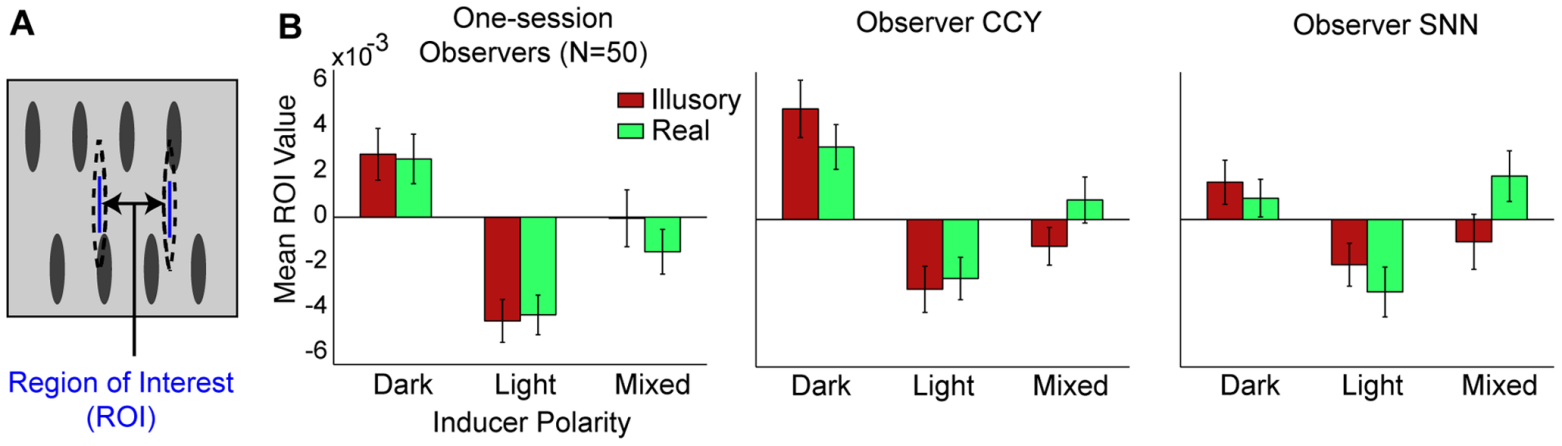
Region of interest (ROI) analysis for the CIs of Experiment 1. (A) To quantify the effects of lightness induction on shape discrimination, slivers of CI pixels were averaged across subjects for one-session observers and across sessions for each multi-session observer. (B) Mean ROI values are shown for each contour type and inducer polarity. Errors depict +/− SEM.

CIs computed from all observers are shown in Figure 4. It can be seen that in the absence of lightness induction, there were no noticeable CI features and in the presence of lightness induction, the key regions were employed in the way predicted. To quantify lightness effects, we performed a region-of-interest (ROI) analysis on the “raw” CIs (viz., those which have not undergone blurring or any other processing.) The ROI was centered within the two key regions and did not overlap with physically visible edges (see Figure 5A and the Analysis section of the Methods for ROI dimensions). The average CI pixel value within the ROI strongly depended on inducer polarity for both one-session and multi-session observers (*p*s<.001). This dependence was comparable for real and illusory contours (*p*s>.09). More specifically, for both contour types, the average ROI value was always positive when the inducers were dark, always negative when inducers were light, and intermediate, otherwise.

To further confirm that dominant CI features correspond to lightened or darkened surfaces, we compared human CIs with those produced by an ideal observer, which discriminated on the basis of a statistically optimal decision rule [14]–[15]. As expected, influential CI pixels corresponded to the edges of the inducers in the illusory conditions and also to the edges of the vertical contours in the real conditions. Importantly, the contour-like CI features in the real-mixed condition (which involved white shape contours) were detectable for ideal but not human observers. The feature-less human CIs cannot be ascribed to subjects neglecting the white luminance-defined contours since the signal contrast thresholds (the oval/contour contrast needed for 70% accuracy) were at least as low as they were in the other real conditions and much lower than in the illusory-mixed condition for the single session and multi-session observers (*ps*<.002). In other words, even though subjects clearly took advantage of the additional shape information in the real-mixed condition, their CIs failed to reveal where that information originated. These data suggest that the CI technique can reveal contour characteristics via lightness induction, but may be severely limited in illustrating shape boundaries when lightness induction is absent.

Salient features also appeared *outside* of the key regions and these were not specifically predicted by our hypothesis. For example, the CIs bowed inward more in the dark than in the light contrast polarity conditions, and features of the opposite contrast polarity arose on the interior of the key regions for the real-light condition. Such features were absent in the mixed polarity CIs. This suggests that lightness induction may guide the employment of non-key regions and, more generally, that contour-surface interactions may be considerably more complex than previously suspected. We briefly speculate on the usage of non-key regions in the Discussion.

**Figure 6:**
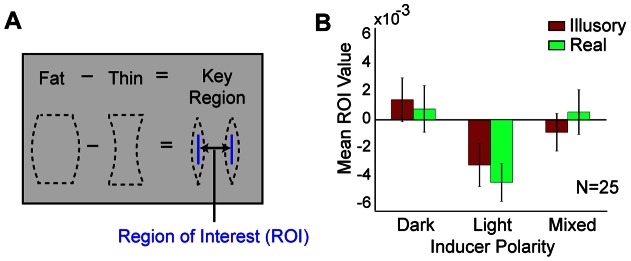
Key region and results for Experiment 2. (A) The shape differences in this experiment were twice as large as the first experiment, but the ROIs spanned the same narrow area. (B) Mean ROI values are shown for each condition. Errors provide +/− SEM.

To show more convincingly that filled-in surface regions alter shape perception, a second experiment was conducted wherein the shape differences were increased so that the key regions were twice as large as before (see Figure 6). The same narrow ROI filter was applied as in the first experiment, so that the analyzed pixels were no longer immediately adjacent to the discriminated contours. Visually meaningful CIs did not emerge because there was one-sixth as much data as in the previous experiment. However, the more sensitive ROI method once again showed a strong effect of inducer polarity, *F*(2, 48) = 7.02, *p* = .002. More specifically, light or dark key region noise pixels tended to be assimilated into the shape when the induced surface color was light or dark, respectively. This effect did not depend on whether the shape was real or interpolated, *F*(2, 48) = 0.51, *p*>.6. These results did not differ appreciably with those found in Experiment 1, *F*(2,146) = 1.04, *p* = .36, indicating that the magnitude of noise influence does not drop off sharply with distance from the discriminated contour.

**Figure 7:**
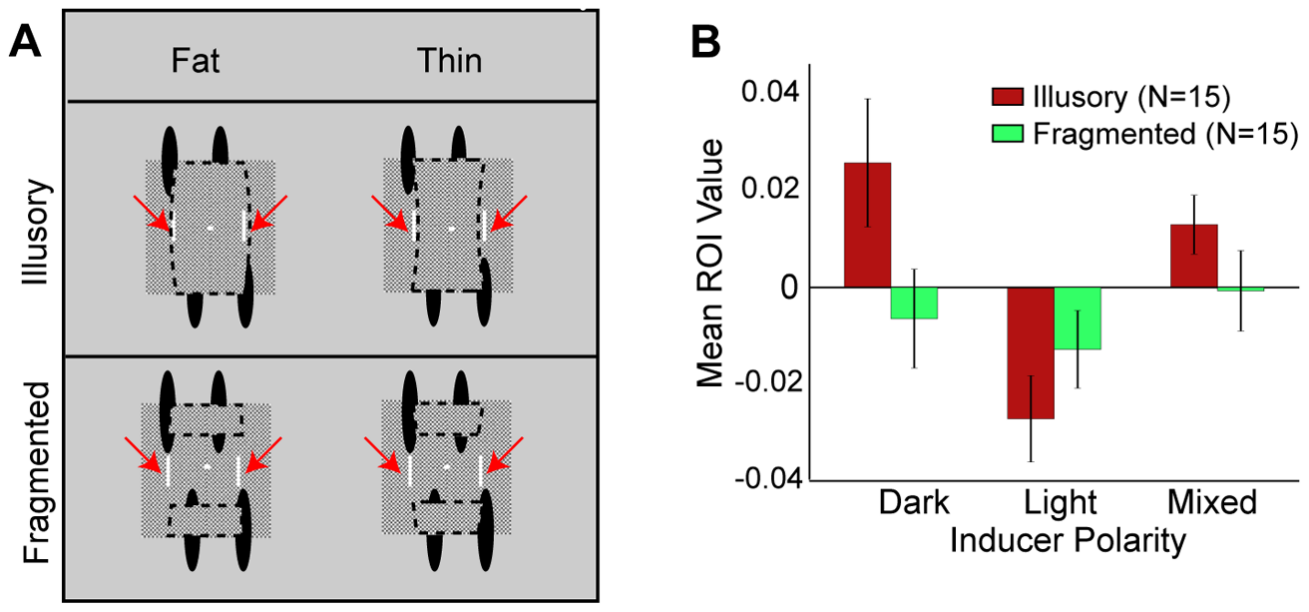
Stimuli and results for Experiment 3 and Experiment 4. (A) Shapes appeared with slender noise bars (identified by red arrows) that were centered within the key regions of the illusory condition. The noise bars were embedded in a fine-grained pattern shown schematically by gray rectangles. The pattern was constant for all trials and appeared under the translating ovals. The top and bottom parts of the fragmented stimuli were trapezoidal and appeared disconnected. (B) A CI was computed with the noise bar values for each subject and ROIs consisted of just the noise regions. Mean ROI values are shown for each polarity condition for the illusory shape discrimination of Experiment 3 and the fragmented discrimination of Experiment 4. Errors are +/− SEM.

A concern may be that the key regions were used only on trials where the noise destroyed the physically visible contours. In these trials, the participants might have been unable to see any shape and would thus be forced to respond only on the basis of surface cues. To better test whether the key regions change shape perception or whether they serve as a shape substitute, we ran a third experiment in which the global noise field was replaced with two slender noise bars centered within the two key regions (see Figure 7A). On each trial, the bars were freshly drawn with a single randomly chosen Weber contrast value. The bars appeared at a fixed location–either immediately outside a thin shape or just within a fat shape. The bars were embedded in a non-varying patterned background so that they would appear as surface features rather than separate objects. Upon computing CIs and performing an ROI analysis on the noise bar regions, inducer polarity once again was found to alter shape perception via lightness induction, *F*(2,28) = 11.5, *p* = .0002. The ROI value was highest when inducers were dark, lowest when inducers were light, and intermediate otherwise (Figure 7B). The effect was strikingly consistent: For all fifteen observers, the ROI value in the dark polarity condition was higher than that in the light, *t*(14) = 5.36, *p* = .0001. The outcome is especially noteworthy in that participants were repeatedly asked to ignore the noise bars throughout each experimental session. The capacity of lightness to alter shape perception apparently cannot be easily abolished through observer strategy, at least not when the inducers are noise-less and high-contrast.

In order to ensure that the foregoing effects depended on visual completion, we conducted Experiment 4, which was otherwise the same as Experiment 3 except that the top and bottom part of the stimulus appeared disconnected (“fragmented” condition; see Figure 7A and Figure S1). The top parts of the fat/thin fragmented shapes were trapezoidal and had the same shape as the top parts of the fat/thin illusory shapes, respectively. The bottom parts of the fat/thin fragmented shapes were identical to the *top* parts of the fat/thin illusory shapes, respectively. The ovals in the fragmented condition were elongated towards the fixation point to further promote a fragmented appearance. The task in the fragmented condition was to indicate via a button-press whether both the top and bottom parts were fat (fuller in appearance) or thin. Here, CI value in the ROI was independent of the inducer polarity, *F*(2, 28) = 0.40, *p* = .67. There was no relationship between noise contrast and observer response because the noise never occurred within a filled-in surface. A comparison to Experiment 3 confirmed the two-way interaction: inducer polarity mattered only if there was an enclosed, filled-in surface, *F*(2,56) = 3.51, *p* = .04. It can be concluded that inducer polarity (oval color) is not enough to explain the CI outcomes from the first three experiments; there must also be a bordered surface that contains the spread of lightness.

Threshold data provide additional confirmation that contour completion occurred in all three illusory conditions of Experiment 3 but not in the fragmented conditions of Experiment 4. Subjects reached threshold accuracy (70%) with lower contrast when the configurations were fragmented rather than illusory, *F*(1,28) = 43.1, *p*<.000001, and this difference did not depend on inducer polarity, *F*(2,56) = 0.34, *p*>.7. In each case, the noise bars likely disrupted visual completion, which in turn made global shape differences harder to see. This finding is consistent with prior studies, which suggest that distractor lines impair discrimination when appearing near completed rather than fragmented shape boundaries [16]–[19]. It can be concluded that contours (but not surfaces) were filled-in regardless of polarity in our spatiotemporal illusory displays.

## Discussion

Our results show that, under noisy viewing conditions, the filling-in of surface color alters perceptually completed shape. More precisely, when the key region noise and the filled-in surface color had the same achromatic color (both light or both dark), the key regions were incorporated into the shape and the shape appeared thin. When the key region noise and surface were of opposite color (one light and one dark), the key regions were excluded from the surface, and the shape appeared fat. When the discriminated shapes were neither darker nor lighter than the background, the noise lost its influence. This pattern was robust. It occurred when shape boundaries were interpolated or real, when the signal was noisy or noise-free, and when the noise was immediately adjacent to or further removed from the fat/thin contours. The effects occurred at a variety of inducer contrast levels (ranging from ∼40% in Experiment 2, to ∼70% in Experiment 3), and could not be abolished by observer strategy. To our knowledge, our experiments are the first to show that–under noisy viewing conditions–lightness induction alters two-dimensional shape perception.

### Reinterpreting CIs and behavioral receptive fields

The present results account for certain currently unexplained aspects of CIs. Referring back to Gold et al.'s (2000) illusory condition, the reason why only two prominent CI stripes appeared on each side of the shape (see Figure 1A) is because there were only two key regions on each side. The reason why these stripes did not obviously bow inward or outward in the illusory condition is because the key regions bowed neither inward nor outward (though see below). Finally, the reason why CI pixels were dark in their study is because–opposite to ours–the first response type was thin (i.e., the subtraction sequence in the classification image equation was reversed; personal communication with J. Gold, September 10, 2006), and noise pixel contrast negatively correlates with (or biases) a thin response when appearing near a lightened shape. Our explanation implies that prominent CI features arising from a fat/thin task do not directly reveal behavioral receptive fields of perceptually completed contours as often supposed [7]–[8], [20]; rather, they correspond to filled-in surface regions *delimited by* contours. That is, completed (and real) contours define the regions in which prominent CI features appear.

### Implications for mechanisms and methodology

An intriguing finding was that–both in our study and in that of Gold et al. (2000)–features of opposite polarity collected on either side of the key regions. In Gold et al.'s illusory condition, for example, the prominent CI features were flanked by areas slightly lighter than the background (see Figure 1A). The flanking areas may modulate the appearance of key regions via lightness induction. Specifically, a key region surrounded by dark pixels will appear lighter than usual, making a fat response more likely for a dark inducer display. The same region surrounded by light pixels would appear darker than usual, rendering a thin response more likely in the same display. The opposite pattern of biases would occur with light inducers. This explanation, while speculative, fits with the other lightness effects documented so far. Of course, this does not mean that key regions *require* the flanking noise to exercise their influence; Experiment 3 demonstrated that key region noise, by itself, can selectively incline a response. But such effects *do mean* that the interaction between lightness and shape perception is not limited to the key regions and that other parts of the stimulus also play a role.

Another observation from the CIs is that the noise near inducers appeared to be more influential than noise in between the inducers. This finding is expected since it has been found in other studies [2], [5], [9] and since physically visible edge information must be relied upon to some extent for above-chance performance. What makes fat/thin CIs so interesting, in our view, is that the filling-in regions are employed at all; that they guide shape perception despite being objectively task-irrelevant.

Our CIs also elucidate the relation between lightness induction, modal completion (for illusory contour formation) and amodal completion (for the representation of occluded contours). Gold et al found pixelwise correlations along the amodally completed edges of fat and thin shapes. Our data imply that the amodally completed surfaces were subject to lightness induction. Moreover, the CI features of Gold et al.'s amodal condition appeared slightly fainter than those of their “illusory” condition. This could be random variation or more likely it could mean that amodally completed surfaces are lightened to a lesser extent than modal surfaces. Another possibility is that–on select trials–the noise rendered the amodal stimulus similar in appearance to the modal stimulus, unleashing lightness induction effects that would otherwise be contained.

While contrast polarity is known to change the grouping strength of contour elements [10], [21] and the depth stratification and phenomenology of completed surfaces [22]–[23], our study is the first to show that perceptually completed 2d shape depends on the color of the filled-in surface, at least under noisy viewing conditions. There are a number of models of illusory shape perception [23]–[25] but none imply this sort of surface-to-shape interaction. The present results give reason to revise the foregoing models.

The neural mechanisms mediating surface-contour interactions remain to be specified, but likely involve recurrent feedforward and feedback connections between low- and higher-tier laminar cortical areas. One possibility is that polarity-sensitive cells of V1 – layer 3B or layer 3 [26] – represent inducing edges. This information is pooled and passed up to the polarity-insensitive layers 2/3 of V2 [23]. These layers, in turn, interpolate contours via excitatory long-range horizontal connections between cells of similar orientation tuning. Illusory contours, once formed, prompt earlier polarity-sensitive cells to become responsive to surface features such as achromatic color, the representation of which depends on lightness induction. When these surface features change, this information is passed up again and prompts the visual system to “re-draw” the interpolated boundary. CIs of dynamic real and interpolated shapes provide indirect evidence for such interactions. For example, in Keane et al., (2007) [9], when different parts of a moving contour appeared at different points in time and when a CI was computed for each frame of motion, luminance noise began to influence shape perception before a completed contour could appear (p.3470, especially frame 2 of the illusory and real conditions), and continued to affect shape judgment well after the contour appeared (see frame 5 of the same page). Evidence for early and late influence of luminance noise on illusory shape perception has also been adduced elsewhere [5]. Such results imply that there exists a continual dialogue between surface and contour forming mechanisms, where the representations formed by one kind of mechanism inform and update the representations formed by the other.

The present findings recommend ways to extend the CI methodology. First, and perhaps most obviously, fat/thin CIs can objectively measure the lightness of perceptually completed shapes. Questions regarding how surface lightness varies with surround contrast [27], variability of inducer polarity [28], temporal frequency [29], or attention [30] can all be objectively addressed by examining the magnitude and direction of noise correlations. Second, classification imaging, which is dogged by notoriously low power, can be applied more efficiently by inserting noise bars within or averaging over key region pixels. In the illusory condition of Experiment 3, for example, we found a main effect of inducer contrast polarity after having run only 1200 trials per condition (6 subjects), which is almost an order of magnitude better than previous studies [2], [5], [9]. Finally, our results show for the first time that the signal does not need to be corrupted with noise for a classification image to be computed; only what the visual system *treats* as signal needs to be corrupted, which in our case is the filling-in regions.

## Conclusion

By having explained seemingly inconsequential CI characteristics, we uncovered a new and robust causal relation that holds between lightness induction and perceptually completed shape. Our results also give a new interpretation of behavioral receptive fields, and offer a constraint on existing models of object perception. Despite these advances, we, again, do not claim to have explained all CI characteristics. For example, in our study (and in Gold et al.'s), the CI contours bowed inward in the dark real condition. The difference may ultimately owe to asymmetries in lightness and darkness induction (see pp.543–544 in [31]) or to poorly understood interactions between luminance-defined contours and lightness. Future studies will need to investigate these CI subtleties to further illuminate how contour and surface forming mechanisms interact to generate representations of complete objects.

## Methods

### Ethics Statement

This study was conducted in accordance with the Declaration of Helsinki. All participants provided written informed consent. The study was approved by the UCLA Institutional Review Board and the Hong Kong University Institutional Review Board.

### Observers

In 4 experiments, the number of subjects included (and excluded) was: 52(5), 25(2), 15(2), and 15(0). Two of the excluded subjects in the first experiment were multi-session (aiming to complete 60,000 trials). Inclusion in the first two experiments required reaching 70% accuracy with maximum contrast; inclusion in the last experiments required performing above chance with maximum contrast. All subjects reported normal or corrected-to-normal vision and were naïve to the purposes of the task. Monetary compensation or class credit was granted for participation. Observers provided written consent to participate.

### Apparatus

The displays were achromatic and presented on a gamma corrected CRT monitor with a resolution of 1024×768 pixels. The background luminance was 37 cd/m^2^. Displays were programmed in MATLAB using Psychophysics Toolbox [32], [33]. In the first two experiments, the refresh rate was 85 Hz, and observers were seated with a chinrest about 121.6 cm from the screen (display  = 18.7×14 deg; pixel = 0.018 deg). In the remaining two experiments, the chinrest was about 243 cm away (display = 9.3×7 deg; pixel = 0.009 deg) and the refresh rate was 100 HZ.

### Stimuli

#### Stimuli of Experiment 1

There were eight ovals, half of which appeared on top and the other half on the bottom. The oval movement direction was randomly chosen on each trial to be either left-to-right or right-to-left. Such randomization reduced the possibility of anticipatory eye movements and allowed the CI results to generalize across movement types. Similar to prior studies, the ovals were misaligned so that different inducing edges of a fat or thin contour appeared at different points in time [9], [12]. (Pilot data also suggest that this misalignment contributes to contour filling-in; [11]). There were three contrast polarity conditions, corresponding to the color of the ovals. In the dark and light conditions, the ovals were dark or light, respectively. In the mixed condition, two dark ovals were to the right (or left) of two light ovals on the top, and the opposite ordering occurred on the bottom (see Figure 2 and Movies S1–S12). Alternating contrast polarity between, rather than within, ovals has advantages. If *each* oval were half dark and half light, then there would be an additional contour bisecting each oval, and the perceptual organization and spatial frequency structure of the stimulus would change. Varying contrast polarity within ovals would also increase how often an inducing edge alternates from light to dark within a trial, which could conceivably lead to lightness induction [28]. Other aspects of the ovals (size, spacing, and speed) and the justification for those aspects are described elsewhere ([9], Experiment 1, p.3464).

The luminance noise fields in Experiment 1 were highly similar to those of Keane et al. (2007, pp.3464–3466 [9]); certain details are worth repeating. The noise followed a Gaussian distribution, which was truncated to ±2 standard deviations from the background luminance (as in [34]). The noise was freshly presented on each trial, remained constant within a trial, and appeared exactly when the ovals moved. The noise field measured 78 arcmin vertically so that it coincided with the peripheral horizontal borders of the figures. Three aspects of the noise in the current study differed from that of Keane, Lu, & Kellman, 2007 [9]. First, the RMS contrast was reduced from 15% to 10% (before truncation) so that observers could reach threshold accuracy. (Pilot data suggested that the 15% value was too high for a number of observers.) Second, the noise field was mirror symmetric about the central vertical axis; this reduced the CI dimensionality (the number of pixelwise correlations) and therefore improved the CI signal-to-noise ratio [35]. Finally, the noise field was extended horizontally by 31% to accommodate for possible pixel correlations well outside the fat figure's boundaries. This change was made as an exploratory measure to consider whether these peripheral regions were task relevant.

The fat and thin figures were virtually identical to those of Keane et al. (2007, p.3464, [9]). The most significant difference is that the added real contour fragments were the same contrast as the inducers in the dark and light polarity conditions and were the same contrast as the light inducers in the mixed condition. The decision to draw the added line segments as white in the mixed polarity condition was arbitrary, but doubtfully had any bearing on the conclusions. Shapes in the real condition were defined by luminance lines (rather than colored surfaces) to follow with precedent [2], [5], [9].

#### Stimuli of Experiment 2

These stimuli were similar to the first experiment, except for the magnitude of the fat/thin shape difference. (See Movies S13–S16). More specifically, the largest horizontal span between physically visible points of a fat shape minus the smallest such span for a thin shape was 8.5 arcmin in Experiment 1 and 17 arcmin in Experiment 2.

#### Stimuli of Experiments 3 and 4

Noise bars replaced the global noise. These noise bars were centered within the key regions, had a length of 26 arcmin, and a width of 1.6 arcmin. The bars were drawn with a single contrast value, which derived from a Gaussian distribution with a RMS of 0.1. To make the noise bars blend in with the background (and appear as surface features), each pixel of the noise bars was embedded into a fine-grained, checkered pattern. Elements that composed the pattern had a Weber contrast of +/− 50%, and a width of 1 arcmin on a side. The pattern completely encompassed the discriminated shapes, always appeared behind the translating ovals, and was the same for all trials (See Movies S17–S20). To make the task sufficiently challenging, we reduced the number of ovals from 4 to 2, increased the oval speed to 9.9 deg/s (instead of 8.4 deg/s), and decreased the physically visible shape differences between fat and thin shapes to 4 arcmin. The angular size of the ovals and the vertical/horizontal dimensions of the rectangles were the same as before. The fragmented stimuli in the last experiment were the same as the illusory, except that the upper portion of the lower ovals and the lower portion of the upper ovals were extended in length by 7 arcmin. The elongation caused the centers of the oval to be shifted centrally (since the peripheral tips of the ovals remained at the same location.) The elongated ovals never overlapped with the noise bars and contributed to the disconnected appearance between the top and bottom parts of the figure. Also, the bottom visible fragment was a translated version of the top so that the top and bottom edges were always misaligned (similar to the fragmented condition of Keane et al., 2007 [9]; see also Figure 7 and Figure S1).

### Procedure

In Experiment 1, subjects had to indicate with a button-press whether a rectangle was fat (bulging outwards) or thin (tapering inwards). All subjects were informed that it was important to maintain fixation on a central point throughout a trial. Each session consisted of six blocks of trials and lasted 45–60 minutes. Three consecutive blocks contained luminance-defined contours and another three consecutive blocks contained illusory contours. The ordering of the contour types was counterbalanced. Each half of a session had a block of white, dark, and mixed inducer trials, and these three blocks were randomized. Two “multi-session” observers each performed 40 1500-trial sessions (250 trials/block). Fifty additional one-session observers each performed 1200 trial in a single sitting (200 trials/block). One-session observers always began with 40 trials of practice. Thus each multi-session observer performed 10000 trials/block, and the one-session observers jointly performed 10000 trials/block. A Bayesian adaptive staircase (QUEST) maintained 70% performance by altering oval (and, if applicable, line segment) contrast from trial to trial [13]. The procedure for Experiment 2 was the same as that of the one-session observers in Experiment 1. In Experiment 3 and Experiment 4, each observer began with 100 trials of practice, and then received 200 trials for each of the three polarities. Polarity ordering was counterbalanced between observers. The task in Experiment 4 was to indicate whether the top and bottom pieces (trapezoids) were fat (fuller in appearance) or thin (trimmer in appearance). Also, participants were given diagrams prior to the experiment indicating where the noise bars would appear. Participants were reminded at the beginning of each of the four blocks (including practice) to ignore the bars when discriminating the shapes.

### Analyses

CIs were produced by averaging the noise fields for each of the four stimulus-response categories, summing the noise fields associated with the first response type, and subtracting the two remaining averaged noise fields [3], [4]. Fat was (arbitrarily) designated the first response type. This means that light CI regions correspond to pixels where noise contrast positively correlates with a fat response, and dark CI regions correspond to noise contrast that negatively correlates with a fat response. For the ideal observer simulations, oval contrast was adjusted so that accuracy was at 70% for 3000 trials. On each trial, the ideal observer utilized a Bayesian decision rule to determine whether the noisy stimulus was more likely a fat or thin shape template (see ideal observer section below).

Only certain parts of the classification images were analyzed; these were jointly termed the “regions of interest” or ROI. In the first two experiments, the ROI was 3 arcmin wide on each side, which is the same as the width of the largest gap between the fat and thin visible portions of the shape in experiment 1. The ROI was centered within the key regions, and measured 39 arcmin vertically. CI pixels falling within the ROI were averaged across subjects for the group of 50 one-session observers and across sessions for each multi-session observer (SNN, CCY). Classification images were blurred with a convolution kernel that was the outer product of [1 2 3 2 1]^T^; other details of the blurring were the same as Keane et al., 2007. All ROI analyses were performed on raw (unprocessed) CIs. In the last two experiments, classification images were computed with just the noise bar values, and ROI analyses were performed on just the noise bar regions of the raw CIs.

#### Ideal observer

An ideal observer is derived by comparing the stimulus input with the noise-free signal image frames, i.e.; the templates for fat and thin figures presented in piecemeal fashion over time. This ideal observer adopts the Bayesian inference framework to make optimal decisions using available information. In our discrimination experiment, a dynamic stimulus input **I** was generated from superimposing two matrices: a noise field **N** in which each noise pixel is independently sampled from a Gaussian distribution with mean 0 and variance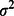; and one of the two templates(**F, T**) representing the targets for the fat and thin figures. As a result, the noisy stimuli **I** can be written as
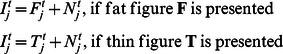
where 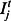, 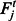,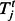, and 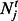 respectively indicate the intensity values of the *j*-th pixel in the *t*-th frame for the stimulus, fat and thin targets, and the noise field, respectively. Given that the stimulus generation is known, we can compute the likelihood probability that a stimulus appears to be a fat or a thin figure as,



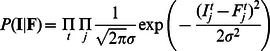


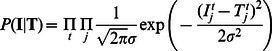



The decision rule of the ideal observer is to compare the two likelihood probabilities and choose the template target that provides the maximum likelihood.

## Supporting Information

Figure S1 **Dimensions of shapes and ovals in Experiments 3 and 4.** The fragmented and illusory stimuli were closely matched on most dimensions.
(TIF)

Movie S1 **Movie of fat illusory rectangle with dark inducers.** In all movies, the shapes are presented in high contrast and the luminance noise is presented in reduced contrast for illustration purposes. Movies play in QuickTime, and are optimally viewed from about 4 feet (Experiments 1–2) or 8 feet (Experiments 3–4), assuming a screen pixel size similar to that described in the Methods.
(MOV)

Movie S2 **Movie of thin illusory rectangle with dark inducers in Exp 1.**
(MOV)

Movie S3 **Movie of fat illusory rectangle with light inducers in Exp 1.**
(MOV)

Movie S4 **Movie of thin illusory rectangle with light inducers in Exp 1.**
(MOV)

Movie S5 **Movie of fat illusory rectangle with mixed inducers in Exp 1.**
(MOV)

Movie S6 **Movie of thin illusory rectangle with mixed inducers in Exp 1.**
(MOV)

Movie S7 **Movie of fat real rectangle with dark inducers in Exp 1.**
(MOV)

Movie S8 **Movie of thin real rectangle with dark inducers in Exp 1.**
(MOV)

Movie S9 **Movie of fat real rectangle with light inducers in Exp 1.**
(MOV)

Movie S10 **Movie of thin real rectangle with light inducers in Exp 1.**
(MOV)

Movie S11 **Movie of fat real rectangle with mixed inducers in Exp 1.**
(MOV)

Movie S12 **Movie of thin real rectangle with mixed inducers in Exp 1.**
(MOV)

Movie S13 **Movie of fat illusory rectangle with dark inducers in Exp 2.**
(MOV)

Movie S14 **Movie of thin illusory rectangle with dark inducers in Exp 2.**
(MOV)

Movie S15 **Movie of fat real rectangle with dark inducers in Exp 2.**
(MOV)

Movie S16 **Movie of thin real rectangle with dark inducers in Exp 2.**
(MOV)

Movie S17 **Movie of fat illusory rectangle with mixed inducers in Exp 3.**
(MOV)

Movie S18 **Movie of thin illusory rectangle with mixed inducers in Exp 3.**
(MOV)

Movie S19 **Movie of fat fragmented figure with mixed inducers in Exp 4.**
(MOV)

Movie S20 **Movie of thin fragmented figure with mixed inducers in Exp 4.**
(MOV)
