# Supplementary figures and images for: Reinterpreting Behavioral Receptive Fields: Lightness Induction Alters Visually Completed Shape
Source: PLoS One. 2013 Jun 4;8(6):e62505. doi: 10.1371/journal.pone.0062505 (PMC3672097; doi:10.1371/journal.pone.0062505)

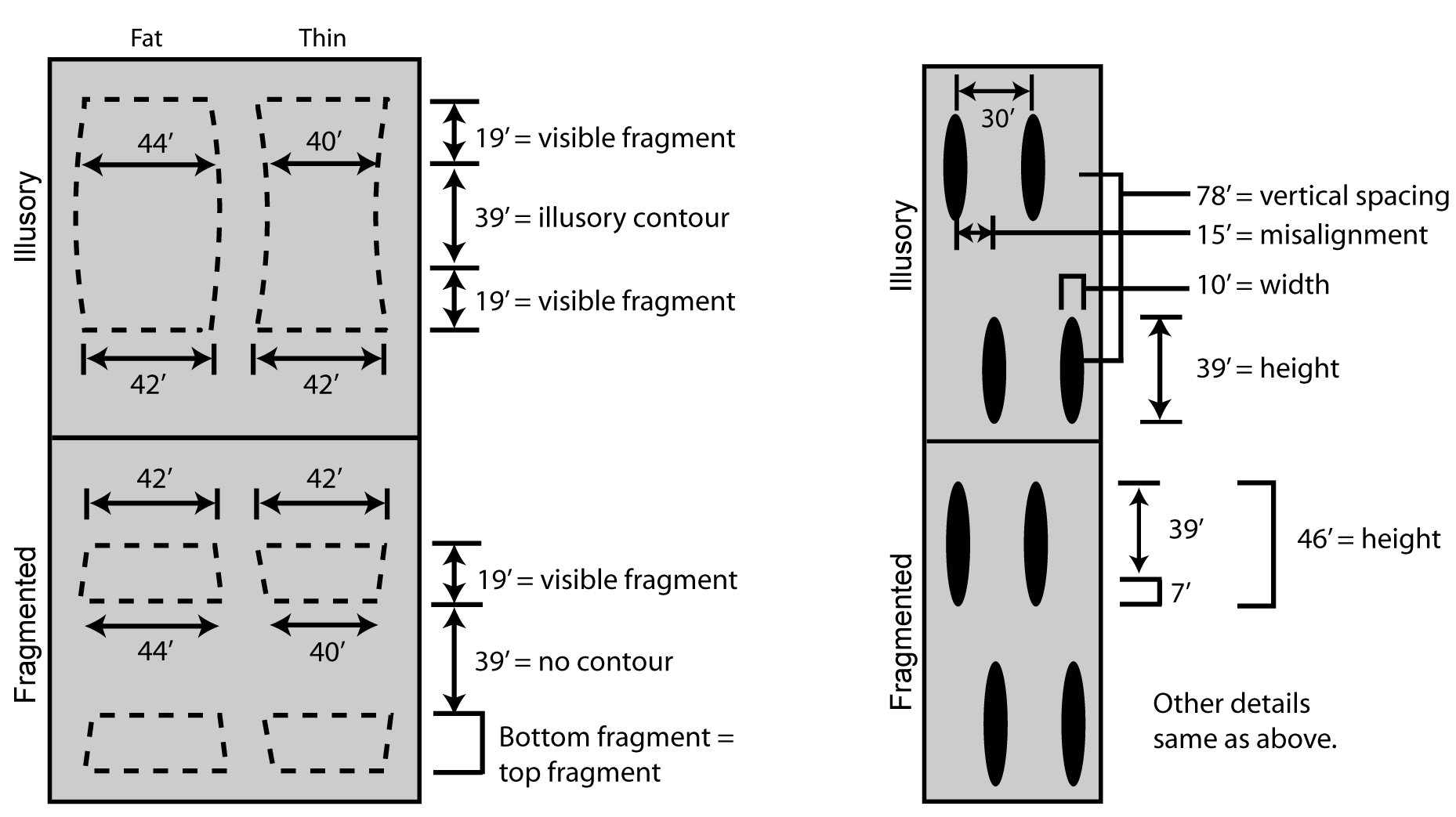

Supplement: Figure S1 — Dimensions of shapes and ovals in Experiments 3 and 4. The fragmented and illusory stimuli were closely matched on most dimensions. (TIF) [file pone.0062505.s001.tif]
